# Response to Revisiting: “Prevalence of and factors associated with sarcopenia among multi-ethnic ambulatory older Asians with type 2 diabetes mellitus in a primary care setting”

**DOI:** 10.1186/s12877-021-02563-6

**Published:** 2021-12-11

**Authors:** Foon Yin Fung, Ngiap Chuan Tan

**Affiliations:** 1 MOH Holdings, 1 Maritime Square, #11-25 HarbourFront Centre, Singapore, 099253 Singapore; 2 SingHealth Polyclinics, SingHealth-Duke NUS Family Medicine Academic Clinical Programme, 167 Jalan Bukit Merah, Connection One, Tower 5, #15-10, Singapore, 150167 Singapore

**Keywords:** Sarcopenia, Asian, Type 2 diabetes mellitus, Primary care

## Abstract

To address and clarify the concerns of fellow researchers of “**Revisiting: “Prevalence of and factors associated with sarcopenia among multi-ethnic ambulatory older Asians with type 2 diabetes mellitus in a primary care setting”**. While chronic obstructive pulmonary disease may be a contributing factor, it is our study limitation to capture a significant number of patients with chronic obstructive pulmonary disease for analyses. Also, ethnicity is not associated with sarcopenia.

## Main text

Sarcopenia is the age-related loss of muscle mass and muscle function, a clinical syndrome associated with increased disability, falls, hospitalization and mortality [1–3]. The loss of muscle mass has been attributed to aging, underlying inflammation, endocrine dysfunction, insulin resistance, nutritional deficit and physical inactivity [4].

Skeletal muscle dysfunction is observed in people with chronic respiratory disease. Among people with chronic obstructive pulmonary disease (COPD), common manifestations of the muscular system include quadriceps weakness, atrophy and changes in the muscle fiber morphology [5]. These changes suggest that COPD may be associated with sarcopenia. However, a systematic review on sarcopenia in patients with COPD showed that only two studies reported an association between sarcopenia and COPD [6]. It also indicated that about 22% of individuals with COPD had sarcopenia, with prevalence rates varying between 12.4 and 28.1% in clinical settings, and 7.9 and 8.4% in population-based settings. Prevalence was highest in nursing home settings, ranging from 53.8 to 66.7%. However there was a large heterogeneity across studies due to differences in methods and cut-off points used to assess muscle mass, and particularly the distinct population settings [6]. Another study alluded to high levels of systemic inflammation in patients with COPD and sarcopenia, though the inflammatory mechanisms are currently unknown [7].

We thank Li et al. for their keen interest in our paper, “Prevalence of and factors associated with sarcopenia among multi-ethnic ambulatory older Asians with type 2 diabetes mellitus in a primary care setting” [8].

We have stated our exclusion criteria clearly: “Those with known risks which hindered or compounded sarcopenia assessment, such as history of stroke, carpal tunnel syndrome, severe hip or knee osteoarthritis, dysarthria or dysphasia, hearing difficulties, use of walking aid, physical disabilities that affect hand-grip and/or walking, use of electronic implants such as pacemaker, and living in residential care facilities were excluded. Patients with any form of other disabilities, such as cognitive impairment, which rendered them incapable of providing informed written consent were also excluded” [8].

We recognize the potential factors associated with sarcopenia due to other comorbidities, such as hypertension, hyperlipidemia, ischemic heart disease, chronic kidney disease, anemia and others and included these in our analyses. As detailed in Table 1 of our manuscript [8], 26.7% of sarcopenia group and 5.4% of severe sarcopenia had other comorbidities which included but were not limited to asthma, osteoarthritis, COPD etc. We apologize for not being explicit in listing all the other less common comorbidities in our study population, which included COPD. In the univariate analyses, we found that chronic kidney disease and anemia were also associated with sarcopenia but these factors were not found in multivariate analyses.

Based on data from the Singapore Longitudinal Ageing Studies on 2479 Chinese participants aged 55 and above, the prevalence of COPD among Chinese is 26% [9]. Nevertheless, no current data is available to shed light on prevalence of COPD in other ethnic groups in Singapore. COPD could be under-diagnosed or under-reported in local setting. Only 2% of diagnosed patients is estimated to carry a COPD diagnosis based on airflow limitation [10].

The major risk factor for COPD is tobacco smoking. Unlike China where the prevalence of smoking is 27.7% in 2019, the prevalence of smoking in Singapore is estimated to be lower by about half at about 12–14% [11]. Furthermore, local patients with severe COPD are more likely to be managed by pulmonologists rather than by primary care physicians. The setting of our study was in a primary care clinic (polyclinic). Our study was limited by a small population of patients with both T2DM and COPD. It is not adequately powered to determine the ethnic risk of patients with T2DM, COPD and sarcopenia. A study with a larger study population recruited from both primary and tertiary care settings is needed to evaluate the association between ethnicity, COPD and sarcopenia.

Unlike the homogenous Han ethnic majority group in the population in China, the local population in Singapore consists of multi-ethnic Asians. While Chinese is the major ethnic group, we had recruited other minority groups such as the Malays and Indians. The study provided an opportunity to assess ethnicity as a potential factor influencing sarcopenia based on a secondary hypothesis. While univariate logistic regression analyses showed that Chinese ethnicity was associated with greater risk of sarcopenia, multivariable logistic regression did not allude to the same result as reported in the study findings. Likewise in a study on the prevalence and factors associated with sarcopenia among community living elderly with type 2 diabetes in primary care clinics in Malaysia, which is also a multi-ethnic Asian society, no significant association was found between ethnicity and sarcopenia [12]. We did not draw the conclusion that Chinese ethnic groups were associated with a greater risk of sarcopenia among older patients with Type 2 diabetes mellitus (T2DM).

It is important to recognize that the demographic characteristics, living environment, personal lifestyle and behavior differ between populations. It is inappropriate to extrapolate that risk factors observed in a native population are applicable in a foreign population. The Japanese in mainland Japan and those in Hawaii have different cardiovascular morbidity and mortality risks even though they are of the same ethnicity [13]. Fundamentally the epidemiology of a clinical entity can differ across populations in various geographical localities. Failure to recognize this fundamental principle can also lead to erroneous interpretations and attribution to “inappropriate and unscientific research design”. We would like to reiterate that the earlier published study is based on sound research design. The results are contextualized to the multi-ethnic Asian population in urban Singapore but are limited in its generalizability.

## Data Availability

Not applicable.

## Abbreviations

T2DM: Type 2 diabetes mellitus
COPD: Chronic obstructive pulmonary disease
